# Alkaline phosphatase in clinical practice in childhood: Focus on rickets

**DOI:** 10.3389/fendo.2023.1111445

**Published:** 2023-02-02

**Authors:** Giuseppe Cannalire, Simone Pilloni, Susanna Esposito, Giacomo Biasucci, Anna Di Franco, Maria Elisabeth Street

**Affiliations:** ^1^ Pediatrics and Neonatology Unit, University of Parma, Guglielmo da Saliceto Hospital, Piacenza, Italy; ^2^ Unit of Pediatrics, Department of Medicine and Surgery, University of Parma, Parma, Italy; ^3^ Department of Medicine and Surgery, University of Parma, Parma, Italy; ^4^ Department of Laboratory Medicine, Guglielmo da Saliceto Hospital, Piacenza, Italy

**Keywords:** alkaline phosphatase, rickets, differential diagnosis of rickets, bone metabolism, osteoblastic activity

## Abstract

Serum alkaline phosphatase (ALP) and its isoenzymes reflect bone metabolism: ALP increases the ratio of inorganic phosphate to pyrophosphate systemically and facilitates mineralization as well as reduces extracellular pyrophosphate concentration, an inhibitor of mineral formation. On the contrary, low ALP activity is associated with reduction of bone turnover. ALP includes four isoenzymes depending on the site of tissue expression: intestinal ALP, placental ALP, germ cell ALP and tissue nonspecific ALP or liver/bone/kidney ALP. The bone isoenzyme (B-ALP) is involved in bone calcification and is a marker of bone turnover as a result of osteoblastic activity. ALP and its isoenzymes are crucial in the diagnostic process of all the forms of rickets.The most common cause of rickets is vitamin D nutritional deficiency. The aim of this review is to update on the role played by ALP serum concentrations as a relevant marker in thediagnosis and treatment of rickets. Indeed, the diagnosis of rickets is based on its clinical, radiological and laboratory characteristics. An elevated ALP level is one of the markers for the diagnosis of rickets in children, though it is also associated with bone formation process. ALP is also useful for the differentiation between rickets and other disorders that can mimic rickets because of their clinical and laboratory characteristics, and, together with other biochemical markers, is crucial for the differential diagnosis of the different forms of rickets. Age, severity and duration of rickets may also modulate ALP elevation. Finally, ALP measurements are useful in clinical and therapeutic follow-up.

## 1 Introduction

ALP is highly expressed in the cells of mineralized tissues and plays a critical function in the synthesis of hard tissues; it increases the ratio of inorganic phosphate to pyrophosphate systemically, facilitates mineralization and reduces extracellular pyrophosphate concentration, an inhibitor of mineral formation process (1).

The detection of elevated ALP serum levels is pivotal for the diagnosis of rickets; however, ALP may be also increased in other diseases as: hyperparathyroidism, leukemia, Hodgkin’s lymphoma, congestive heart failure, ulcerative colitis and some hepatocyte disease, such as viral hepatitis. Altered osteoblastic activity in Paget’s disease and in fibrous dysplasia is associated to ALP elevation. Other possible causes of increased ALP include hepatobiliary disease: cholestasis increases the synthesis of hepatocyte ALP and the secretion of its molecular weight form into plasma. Elevated values of ALP may be also found in pregnancy (2).

Mutations affecting the Glycosylphosphatidylinositol (GPI) anchor protein to link ALP to cell membranes may lead to apparent persistently high circulating ALP without evidence of bone disease (3). Moreover, congenital GPI biosynthesis defects have been associated with hyperphosphatasia and intellectual disability, often accompanied by epilepsy (4–7).

On the contrary, low ALP activity is found in congenital hypophosphatasia, hypothyroidism, vitamin C and B12 deficiencies, zinc and magnesium deficiency, and protein malnutrition (2).

Rickets includes conditions characterized by defective mineralization of the growth plate and osteoid mineralization (1). The classification of rickets is based on the main mineral deficiency, and is conventionally defined as calcipenic or phosphopenic (8, 9). Both forms are characterized byreduction of phosphate in the growth plate cartilage (10). Assays of serum ALP and its isoenzymes are crucial for the diagnostic process of all the forms of rickets.

The aim of this review is to update on the role of ALP for the diagnosis of different conditions with a specific focus on the diagnosis and treatment of rickets.

## 2 Alkaline phosphatase

ALP is a metalloenzyme that presents with different isoenzymes, among which the tissue-non specific isozyme (TNAP) is strongly expressed in bone, liver and kidney, and plays a key function in bone calcification. ALP is involved in bone calcification from the very early phases. In particular, TNAP hydrolyzes pyrophosphate and supplies inorganic phosphate to enhance mineralization *via* the production, inside matrix vesicles, of hydroxyapatite crystals that bud from the outer membrane of osteoblasts and chondrocytes. Hydroxyapatite then expands into the extracellular matrix and accumulates within collagen fibrils (11). Circulating ALP activity changes during the different phases of life and development as it is a marker of osteoblast activity, thus increasing during the growing phases of childhood and adolescence when bone mineralization and turnover are higher (12). The growth plate chondrocyte production of ALP during active growth contributes to the higher levels of ALP in childhood and adolescence. As a consequence, ALP serum levels are 1.5-2.5 times higher in children than in adults.

Many different biochemical and immunological methods have been used to discriminate among isoforms, and selective assays are capable of differentiating among the different ALP forms. is based on biological enzyme activity, and is not on direct assay of the protein levels. The common measurement of total ALP is based on the enzyme activity, measured by a colorimetric method. In contrast the B-ALP measurement is an immunological assay with better specificity for enzyme. Isoenzyme percentage of the total can be determined also by heating the sample and measuring residual ALP activity but this is less accurate than the direct measurement. ALP activity can be easily measured in serum, most laboratories provide a reference range, under physiological conditions, between 30 and 120 International Units per Liter (IU/L), irrespective of age, pubertal stage and sex (13). These values refer to circulating ALP, which includes ALP from bone (i.e., the main circulating isoform in children and adolescents), liver, intestine, kidney and placenta during pregnancy.

The study by Zierk et al. (14) showed that female ALP activity is higher at 10–12 years of age with a median (50^th^ percentile) and maximum (97,5^th^ percentile) activity of 240 and 400 IU/L, respectively. Male activity peaks at 13–15 years of age with similar median activity (250 U/L) and higher maximum value (450 IU/L) than the female one. Comparing these results (2.5th and 97.5th percentiles) with the results of the Caliper study (15) we can observe similar reference intervals in the first 6 months of life, in particular for the upper limits however, in the following ages the values ​​are higher than those reported in the Caliper (15) and Turan (12) studies for a consistent period of time.

As previously mentioned, ALP comprises four different isoenzymes depending on the site of tissue expression: intestinal, placental, germ cell and tissue nonspecific or liver/bone/kidney (L/B/K) ALP.

**Figure 1:**
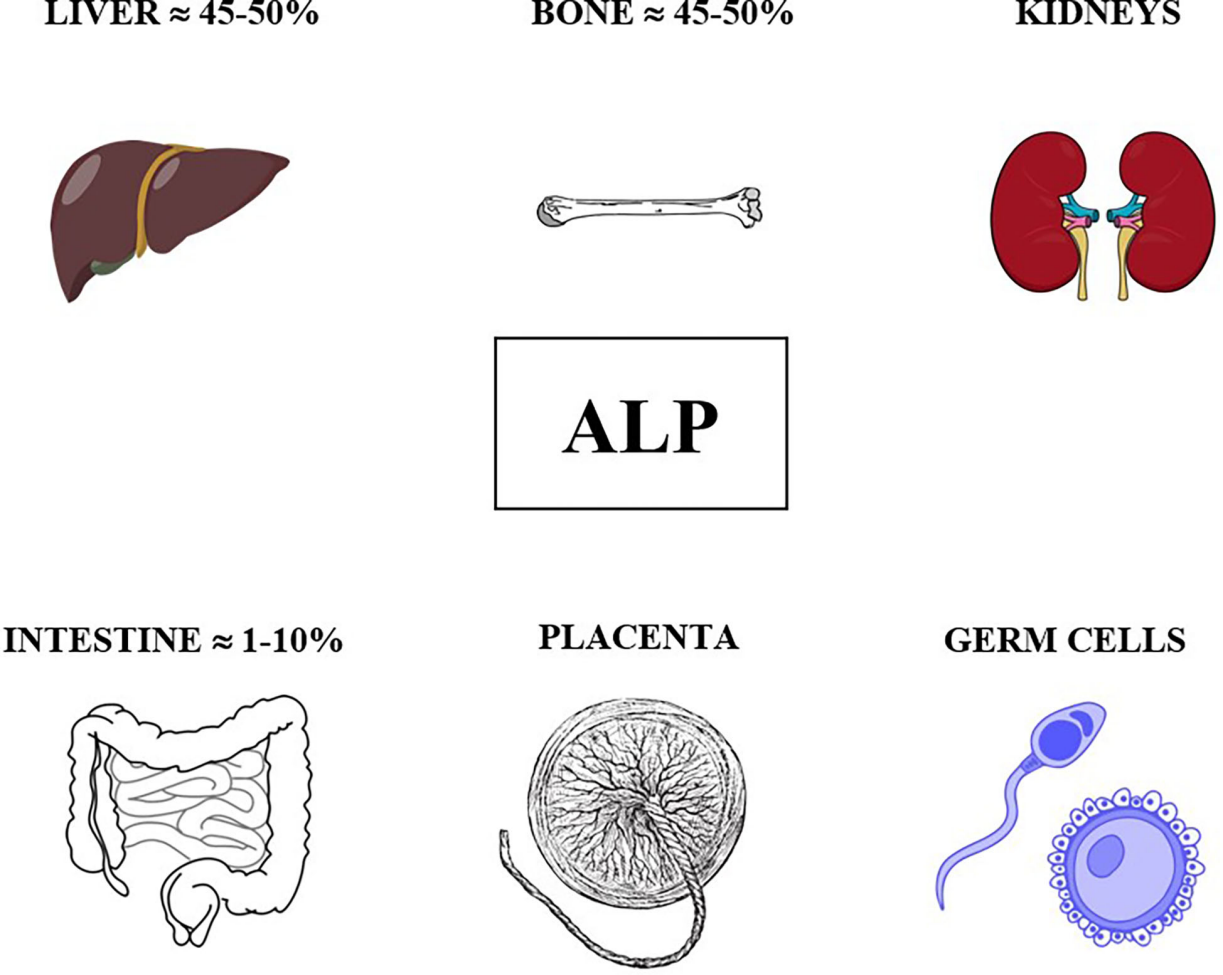
ALP isoenzymes and their circulating levels in healthy children.

ALP isoenzymes and their circulating levels in healthy children are shown in **Figure 1**.

The bone isoenzyme is involved in mammalian bone calcification and the intestinal isoenzyme is thought to play a role in the transport of phosphate into intestinal epithelial cells. Abnormal expression of genetically distinct ALP isoenzymes is valuable in monitoring cancers, particularly germ-cell tumors. On the contrary, subjects with congenital hypophosphatasia have generalized deficiency of L/B/K ALP, with normal values of placental and intestinal ALP isoenzyme.

ALP plays a crucial role in hydrolyzing many phosphate‐containing physiological compounds, thus contributing to DNA synthesis and attenuation of inflammation. For example, the intestinal isoenzyme is fundamental for dephosphorylation of proinflammatory molecules in inflammatory bowel diseases. Finally, ALP is also a useful serum biochemical marker of liver disease, particularly cholestatic disease.

Due to age and sex physiological variations, specific reference ranges are of upmost importance for a correct interpretation of biochemical data in order to suspect and achieve correct diagnoses. Serum total ALP levels are highest during the first 6 months of life, then progressively decreasing to relatively steady levels, and further increasing after 9 years of age, with peak levels during puberty, though not as high as those detected during infancy (13, 15).

## 3 Clinical manifestations of rickets

Most clinical characteristics are present both in calcipenic and phosphopenic rickets. Bone deformities represent the most specific feature of rickets (10). In infants, the site of bone deformity depends on the age at presentation, being related to the forces which bones are exposed to, along with the achievement of infants’ motor developmental milestones (e.g.: crawling, walking, etc.). As a consequence, the most common clinical characteristics involve craniotabes, frontal bossing, delayed fontanelle closure, craniosynostosis, delayed teeth eruption with enamel hypoplasia, progressive bowing deformities of the legs, widening of the wrists and ankles, delayed ability to walk, and a typical waddling gait (8). The progressive bone deformities may also eventually lead to short stature. When rickets occurs later in childhood, legs acquire a typical knock-knee deformity. As rickets signs progress, the clinical picture may also include the characteristic rachitic rosary (i.e. enlargement of the costochondral junctions) and Harrison sulcus (i.e. a groove at the lower margin of the thorax).

Other extra-skeletal manifestations of rickets may include muscle weakness with hypotonia, motor development delay and the lower limbs pain (especially in adolescents) (16). In addition, X-linked hypophosphatemia (XLH) may have dental abscesses and enamel alterations as first clinical signs of the disease (17).

## 4 Biochemical parameters in rickets

The first line biochemical assessment in the rickets diagnostic workup includes serum ALP, in addition to serum calcium, phosphorus, 25OH-vitamin D (25OHD) and parathyroid hormone (PTH) evaluation.

In the case of predominant calcium deficiency, rickets is defined as calcipenic rickets. This form is usually caused by insufficient intake of vitamin D or by the inability to activate vitamin D introduced with the diet. It is important to consider that under some circumstances serum calcium levels can be normal. Calcipenic rickets is then further subdivided into several other conditions based on the measurement of the circulating levels of 25OHD and 1,25(OH)2D3. In case of reduced levels of 25OHD, rickets is usually caused by an insufficient introduction of vitamin D with the diet, and is therefore defined as nutritional rickets. More rare disorders include 25-hydroxylase deficiency. When 25OHD levels are normal, the disorder might be caused by a defect in the function of 1-alpha-hydroxylase or by inherited resistance to vitamin D.

In case of predominant phosphorus deficiency, rickets is defined as phosphopenic. This type of disorder is almost always associated with renal phosphate wasting. The most common types of phosphopenic rickets are the Fibroblast Growth Factor 23 (FGF23) mediated disorders, including XLH rickets, autosomal dominant and autosomal recessive forms of hypophosphatemic rickets (less common) and tumor induced osteomalacia, which may be also present in childhood (17). Other causes of phosphopenic rickets, non FGF23-associated, include renal tubular disorders, hereditary hypophosphatemic rickets with hypercalciuria, and nutritional phosphate deficiency. It is worth noting that 1,25(OH)2D3 is typically high in phosphopenic rickets due to dietary deficiency. In children with calcipenic rickets, the most typical biochemical changes include low or normal serum calcium levels, increased PTH levels and decreased phosphate levels, whereas in hypophosphatemic rickets, serum calcium and 25OHD levels are usually normal, phosphate is low, and PTH levels are normal or only slightly increased (10). If serum levels of PTH, ALP, phosphorus and calcium are normal, the diagnosis of rickets is unlikely.

The role of ALP in the diagnosis of rickets will be further discussed in following sections of this paper.

## 5 Radiologic features

The radiologic signs of rickets can be best identified at the growth plates of those bones with the highest mineral demands, hence those most rapidly growing. Therefore, the most useful radiographic exams to study the distal ulna, and knee X-rays, to examine the metaphyses of the long bones involved in knee joint (10). Typical abnormalities of the metaphyses include cupping, fraying, lateral widening and expansion of the growth plate (10). Another radiographic feature which is often present in rickets is the presence of deformities of the shaft of long bones. Some distinguishing characteristics in case of calcipenic rickets include generalized osteopenia, subperiosteal bone resorption and periosteal reaction along the diaphysis (17). Differently, in case of hypophosphatemic rickets, the involvement of the metaphyses is less evident (18). Metaphyseal fraying, which is a typical finding in calcipenic rickets, may be observed also in hypophosphatemic rickets especially prior to treatment, with wide range of skeletal severity.

## 6 The role of ALP in the diagnosis of calcipenic rickets

The first identification of patients with calcipenic rickets is based on laboratory findings such as elevated serum PTH and normal/low phosphorus, with either normal (especially in the early stages of the disease due to compensatory PTH increase) or low serum calcium levels (19). In calcipenic rickets, ALP levels often reach much higher levels (up to 2000 IU/L) than those commonly observed in phosphopenic rickets. In nutritional rickets the rise in ALP is commonly seen before serum calcium and/or phosphate drop, and it anticipates also the specific radiographic changes, indicating its potential role in the early diagnosis of the disease (20). ALP levels can be markedly elevated also in the case of rarer causes of calcipenic rickets, such as 25-hydroxylase deficiency (i.e., a condition characterized by defects in the enzyme responsible for the 25-hydroxylation of vitamin D). This was demonstrated for instance by Molin et al. (21), in two children who presented with marked hypocalcemia, hypophosphatemia, and high PTH and ALP levels.

As seen in 25-hydroxylase deficiency, the elevation of serum ALP levels is also typical of 1-alpha-hydroxylase deficiency (also defined as vitamin D-dependent rickets type 1 or VDDR1), a somewhat more common condition in which the genetic defect causes a defective conversion of 25OHD into the active form of vitamin D. In the case series published by Dodamani et al. (22), in which the authors reviewed the clinical features of a total of 165 probands affected by VDDR1 found in literature, the patients presented with markedly elevated ALP levels (on average 1480 IU/L). Ozden et al. (23) observed similar findings in their case series including nine patients affected by mutations involving vitamin D 1-alpha-hydroxylase; in this series the ALP values at presentation ranged from 939 U/L to 2363 U/L (with the exception of one patient who interestingly had an ALP value of 255 U/L in a setting of normal biochemical parameters except for hyperparathyroidism).

Increased serum ALP is also part of the biochemical spectrum of children affected by Hereditary Vitamin D Resistant Rickets (HVDRR) (24), another rare form of calcipenic rickets caracterized by the resistance to vitamin D active form, usually due to mutations involving the vitamin D receptor gene.

## 7 The role of ALP in the diagnosis of phosphopenic rickets

Phosphopenic rickets is initially commonly characterized by normal or slightly elevated serum PTH and low serum phosphorus. Renal phosphate wasting is the most common cause of hypophosphatemic rickets (17). The determination of the tubular resorption of phosphorus (TRP) and maximal renal tubular threshold for phosphate per glomerular filtration rate (TmP/GFR) is mandatory to differentiate the latter forms from the ones characterized by renal conservation of phosphate. As reported above, ALP levels are usually increased also in case of phosphopenic rickets (usually ranging between 400 and 800 IU/L), but to a lesser extent than in calcipenic rickets. The most common hereditary form of hypophosphatemic rickets is XLH (23). The increase in ALP levels is one of the biochemical hallmarks of this condition, together with hypophosphatemia, and elevated intact FGF23 levels. For this reason, current guidelines recommend serum ALP measurement for the initial identification of a possible case of XLH (25). Some case series studies available in literature report median serum ALP levels around 500 IU/L at onset in XLH patients (26). Considering the rarer hereditary forms of hypophosphatemic rickets, the biochemical features at presentation of pediatric patients affected by Autosomal Dominant Hypophosphatemic Rickets (ADHR) report ALP levels ranging from 190 IU/L to 1755 IU/L, with most values below 1000 IU/L (27). Increased serum ALP is also seen in the Autosomal Recessive form of Hypophosphatemic Rickets (ARHR), which is further subdivided in three subtypes depending on the gene involved. Höppner et al. (28) reported that total serum ALP was increased in most cases of ARHR type 2 (caused by mutation in the ENPP1 gene) patients, though it might also be found within normal range at the disease onset. The magnitude of ALP elevation is usually limited also in this subtype of hypophosphatemic rickets, as shown in the familial case reported by Choe et al. (29), where the patient with homozygous mutation in the *ENPP1* gene had ALP values of 472 IU/L at presentation, while parents and sister (presenting with heterozygous mutation) had ALP values ranging between 95 IU/L and 200 IU/L. Similar levels of serum ALP elevation were also identified in patients affected by mutations causing hypophosphatemic rickets with hypercalciuria (HHRH), with values 2-4 fold higher than the upper limit of normal reference values (30). Hypophosphatemic rickets can be also caused by nutritional phosphate deficiency. In this condition, renal studies show increased renal conservation of phosphorus. Literature reports similar findings in children exclusively fed with a specific elemental formula (Neocate® amino-acid formula) (31). Furthermore, some case series reports showing increased serum ALP levels (values ranging from 776 IU/L to 3777 IU/L) at disease presentation are available in the literature; in these subjects ALP levels are likely higher that those normally seen in phosphopenic rickets due to hereditary phosphate wasting conditions. ALP values in different forms of rickets and in disorders that mimic rickets are shown in **Table 1**.

**Table 1:** ALP values in different forms of rickets and in disorders that mimic rickets.

CALCIPENIC RICKETS (ALP levels usually up to 2000 IU/L)				
**Nutritional rickets** 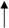	**25-hydroxylase deficiency** 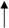 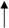	**1-alpha-hydroxylase deficiency** 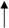 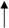		**HVDRR** 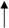 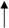
PHOSPHONIC RICKETS (ALP levels usually between 400 and 800 IU/L)				
**XLH** 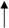	**ADHR** 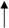 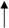 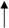	**ARHR** N- 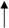	**HHRH** 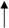	**Nutritional phosphate deficiency** 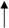 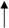
**DISORDERS THAT MIMIC RICKETS**			**TH** 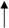 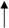 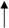	
**Hypophosphatasia** 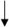 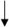				

HDVRR, Hereditary Vitamin D Resistant Rickets; XLH, X-linked hypophosphatemia; ADHR, Autosomal Dominant Hypophosphatemic Rickets; ARHR, Autosomal Recessive Hypophosphatemic Rickets; HHRH, Hereditary Hypophosphatemic Rickets with Hypercalciuria; TH, Transient Hyperphosphatasemia.

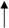
: increased; 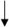: decreased; N: Normal.

## 8 The role of ALP in hypophosphatasia and transient hyperphosphatasemia

Among those conditions that may mimic rickets because of similar signs and symptoms, two pathological entities deserve a special mention: hypophosphatasia and transient hyperphosphatasemia (30–33). Hypophosphatasia is a condition characterized by a reduction of serum ALP levels (30). This is due to mutations in the gene encoding for tissue non-specific ALP. This disease can present at different ages, with the most severe form occurring in the perinatal period. The signs and symptoms resemble those classically seen in rickets (32). The biochemical hallmark of the disease, which is fundamental for the initial differentiation between hypophosphatasia and rickets, is reduced serum ALP. Values lower than 150 U/L in neonates should raise suspicion. These values may vary later in life, particularly during puberty (11). In the most severe form, reduced incorporation of minerals in the skeleton results in elevated calcium and phosphate, with reduced PTH levels and hypercalciuria. Differently from other form of rickets, milder forms show normal mineral homeostasis. It is also worth highlighting that labs only providing adult normal ranges may prevent a correct diagnosis of hypophosphatasia.

ALP may increase in patients with hypophosphatasia treated with enzyme replacement. ALP elevation, in this case, should not lead to unnecessary concern.

Transient hyperphosphatasemia (TH) is a condition characterized by an isolated elevation of serum ALP without any other biochemical and/or radiologic marker of rickets. While detection of TH is usually incidental, it raises the need for further evaluation to rule out a rickets condition. TH is not yet clearly defined, as there is no agreement on the ALP cutoff values for its diagnosis, the age limits, and the possible causes. A possible association between TH and acute respiratory infections has been suggested (33). It is interesting to consider that the ALP levels are usually markedly elevated in TH, sometimes as high as 4000-7000 IU/L (30).

Moreover, elevated ALP, as a byproduct of osteoblast activity during active bone formation, may also be found in Juvenile Paget’s disease and/or in fibrous dysplasia of bone (34).

Juvenile Paget’s disease (JPD) is a focal disorder of bone remodelling that progresses slowly and leads to changes in the shape and size of affected bones, and to skeletal deformity. Extra-skeletal manifestations include hearing loss, retinopathy, vascular calcification and aneurysm formation of internal carotid artery. Phenotype severity seems to be related to the severity of TNFRSF11B gene deactivation. JPD is biochemically characterized by very high ALP levels, as well as elevation of other markers of bone turnover (35).

Fibrous dysplasia of bone is characterized by bone pain, bone deformities and fractures, involving one or several bones. It is caused by missense mutations in the gene encoding for the alpha-subunit of the stimulatory G-protein alpha stimulating (GNAS) complex locus, on chromosome 20q13. This mutation results in osteoblastic differentiation defects, and bone resorption is often increased with elevated ALP. The bone lesions may be associated with endocrine dysfunctions and café-au-lait spots; this is known as McCune-Albright syndrome. Patients with polyostotic fibrous dysplasia often have renal phosphate wasting. The disease, however, presents a wide clinical spectrum (35).

## 9 The role of ALP in monitoring rickets therapy

Serum ALP has also been proposed as a valuable marker of patients’ response to rickets treatment and management (36–38). The regular monitoring of ALP levels during treatment is indicated in case of nutritional rickets. Chatterjee et al. (37) recommended to include ALP in the laboratory monitoring of nutritional rickets treated with high doses of vitamin D, on a stoss therapy regimen. The authors also recommended clinicians to use ALP measurement alone to monitor the biochemical improvement in the follow-up of nutritional rickets, since ALP is a reliable and cost-effective marker of disease progression (37).

In the study by Uday et al. (39), ALP was proposed as an indicator of disease activity in children with XLH on conventional therapy in order to evaluate the response to conventional and Burosumab treatment. The authors, however, stressed the fact that ALP should not be used as the only indicator of disease activity and should be considered together with radiologic signs of disease progression and regression (36).

Indeed, serum ALP seems to be the most useful biomarker for skeletal response in XLH treatment being slightly elevated prior to treatment and decreasing during treatment, thus serving as a surrogate marker for bone healing (38). Finally, the role of ALP levels reduction as a target of effective response to monoclonal antibody to FGF23 in the treatment of XLH rickets has also been determined (40, 41).

It is important to specify that, in multiple rickets conditions, ALP may transiently further rise after initiating treatment, before decreasing to normal.

We hypothesize that the normalization of ALP, especially the bone isoenzyme, may represent a marker of the therapeutic effect for new generation bone therapy, and orthopedic pathologies.

## 10 Discussion

ALP measurement is crucial in the diagnostic process of all forms of rickets. It is worth to stress out the physiological variations of ALP reference levels in the different phases of growth and, therefore, the need to adjust ALP reference values according to patient’s age and sex (42). Overall, it is worth considering how much higher than the upper limit for age the ALP values are, as apparently elevated total ALP levels in some forms of rickets may be due to age related physiologically high values. On average, serum ALP is generally more elevated in the calcipenic (up to 2000 IU/L) than in the phosphopenic forms of rickets. Baseline ALP values might “*per se”* give some hints on the cause of rickets, but the assessment of other clinical, biochemical and radiologic features remains of utmost importance to achieve ultimate etiological diagnosis. ALP measurement is also useful in those disorders that mimic rickets in order to correctly identify and treat them. Finally, there is evidence demonstrating that ALP plays a pivotal role in monitoring the efficacy of rickets treatment, hence its measurement should be always included in the biochemical follow-up of patients under treatment. Nevertheless, as ALP values normalization may be associated to hypercalcemia and hypercalciuria in case of overtreatment of some forms of rickets, we also recommend to evaluate other parameters, e.g. PTH, vitamin 25OHD, 1,25(OH)2D3, calcium and phosphorus levels both in blood and urine, in association to auxological and radiological evaluations, in the follow up of patients with rickets.

## Author contributions

GC and SP drafted the manuscript. MS conceived, designed and supervised this study. SE, GB and MS provided scientific contributions and critically revised the paper. All authors contributed to the article and approved the submitted version.
